# Feeding *Saccharomyces cerevisiae* fermentation products lessens the severity of a viral–bacterial coinfection in preweaned calves

**DOI:** 10.1093/jas/skab300

**Published:** 2021-10-21

**Authors:** Paiton O McDonald, Courtney Schill, Teresia W Maina, Beulah Samuel, Madison Porter, Ilkyu Yoon, Jodi L McGill

**Affiliations:** 1 Department of Veterinary Microbiology and Preventive Medicine, Iowa State University, Ames, IA 50010, USA; 2 Diamond V Mills Inc., Cedar Rapids, IA 52404, USA

**Keywords:** bovine respiratory disease, infection, innate immunity, metabolism, preweaned calves, *Saccharomyces cerevisiae* fermentation products

## Abstract

We have previously reported that supplementation with *Saccharomyces cerevisiae* fermentation products (**SCFP**) ameliorates clinical signs and lung pathology following experimental bovine respiratory syncytial virus (**BRSV**) infection in preweaned dairy calves. The objectives of this study were to determine the effect of SCFP supplementation on the metabolic and endocrine responses, and disease outcome of a viral–bacterial coinfection in preweaned calves. Twenty-seven, 1- to 2-d-old Holstein-Angus cross calves were enrolled in the study; one SCFP calf was removed from the trial during the pre-challenge phase due to complications from nephritis. Calves were assigned to two treatment groups: control or SCFP-treated, base milk replacer with 1 g/d SCFP (Smartcare, soluble formula) and calf starter top dressed with 5 g/d SCFP (NutriTek, insoluble formula). Calves were infected with BRSV on day 21, followed 6 d later by intratracheal inoculation with *Pasteurella multocida* (*PM*). Calves were euthanized on day 10 post-viral infection. Calves receiving SCFP had reduced thoracic ultrasonography scores on day 7 post-viral infection (*P* = 0.03) and a tendency toward reduced scores on day 10 post-viral infection (*P* = 0.09). Calves receiving SCFP also had less severe lung pathology scores at necropsy (*P* = 0.06). No differences between treatments were observed in lung viral loads (*P* = 0.48) or bacterial lung recovery (*P =* 0.34); however, there was a distinction in the lung location for *PM* recovery, with *PM* isolated more frequently from the cranial lobes in SCFP-treated calves, but more frequently from the caudal lobes of control calves. Calves treated with SCFP tended (*P* = 0.07) to have higher serum IL-6 concentrations following the coinfection. Calves treated with SCFP had lower concentrations of serum nonesterified fatty acids and beta-hydroxybutyric acid compared with controls following experimental challenge (*P =* 0.03 and *P =* 0.08, respectively), suggesting metabolic changes favoring growth and development. There were no differences between groups in gene expression of insulin receptor, insulin-like growth factor 1 (**IGF-1**), IGF-1 receptor (**IGF-1R**), growth hormone receptor, or haptoglobin in the liver. Results from this study suggest that supplementing with SCFP may moderate the impact of a respiratory viral–bacterial coinfection on preweaned calves through metabolic and immune modifications.

## Introduction

Bovine respiratory disease complex (**BRDC**) is a multipathogenic syndrome that affects all stages and categories of cattle production. Typically, BRDC is initiated by a primary viral infection that predisposes the animal to exacerbated and frequently fatal, secondary bacterial pneumonia (Taylor et al., 2010). Common strategies to control BRDC include vaccines, antimicrobial, and ancillary therapies. Although widely implemented, the tools currently available to manage BRDC do not always meet the expectations of the cattle industry and there has been little reduction in the incidence of BRDC over the last two decades (USDA, 2010, 2012; Smith et al., 2020). Stressful events such as weaning, shipping, commingling, and administration of vaccines can suppress the capacity of an animal to respond to treatment and preventive practices. With societal concerns mounting over metaphylactic and prophylactic antibiotic use for the prevention of BRDC, there is an increasing need for nonantibiotic treatments that generate broad-spectrum protection from the pathogens that cause BRDC in cattle.

*Saccharomyces cerevisiae* fermentation products (**SCFP**) have positive effects on many aspects of immune function in cattle, swine, poultry, and humans (Moyad et al., 2009; Shen et al., 2011; Brewer et al., 2014; Alugongo et al., 2017). In a previous study, we observed that supplementation with SCFP modulated innate immune function in preweaned calves (Mahmoud et al., 2020). Immune cells isolated from the blood of SCFP-fed calves had an increased capacity for proinflammatory cytokine production, whereas immune cells isolated from the lungs produced fewer proinflammatory cytokines (Mahmoud et al., 2020). Subsequently, calves receiving SCFP developed less clinical disease and less lung pathology following an experimental bovine respiratory syncytial viral (**BRSV**) infection (Mahmoud et al., 2020).

Activation of the immune system is an energy-intensive process. Feedlot steers and dairy cows undergoing stress or immunologic insult are known to reprioritize their metabolic uses and energy stores to help maintain a robust and active immune response (Kvidera et al., 2016, 2017). However, supplementation with yeast or yeast products may have some beneficial effects on energy partitioning and metabolic performance in animals undergoing an immunologic insult. In one recent report, preweaned beef calves supplemented with SCFP had improved clinical scores and metabolic outcomes during a lipopolysaccharide (**LPS**) challenge (Burdick Sanchez et al., 2020). In another example, feedlot heifers supplemented with *S. cerevisiae* had improved metabolic outcomes during a respiratory disease challenge with bovine herpesvirus-1 and *Mannheimia haemolytica* (Word et al., 2019). Like adult cattle, preruminant calves require considerable energy to mount an immunologic challenge. Supplementation with SCFP has been reported to enhance growth in preweaned calves due to glucose partitioning in response to an immune response (Klopp et al., 2020) and increase dietary intake which attributed to enhanced growth (Lesmeister et al., 2004). Therefore, SCFP may provide additional support to the growing animal undergoing an immunologic insult and thus impact the metabolic and endocrine responses of calves responding to respiratory infection.

Our prior study assessed the impact of SCFP on an individual pathogenic infection. However, BRDC is a complex disease often involving multiple pathogens. Calves with BRSV and *Pasteurella multocida* (*PM*) infection often present with a severe pneumonic infection in the lower respiratory tract (Sudaryatma et al., 2020). Therefore, the objective of this study was to determine the impact of SCFP treatment on the outcome of a BRSV-*PM* coinfection in preweaned dairy calves. The secondary objective was to evaluate the metabolic and endocrine responses of preweaned calves to SCFP supplementation following a viral–bacterial coinfection. We hypothesized that SCFP supplementation would improve endocrine and metabolic responses, innate immune responses, and disease severity following experimental BRSV-*PM* coinfection in preweaned calves.

## Materials and Methods

The experimental procedures were approved by the Iowa State University Institutional Animal Care and Use Committee (protocol 20-113) and the Institutional Biosafety Committee (protocol #20-066).

### Animal care and feeding

The study was conducted as a randomized complete block design consisting of one 32-d period. Twenty-seven, 1- to 2-d-old Holstein × Angus calves (12 males and 15 females) were enrolled in the trial. A statistical power analysis was performed for sample size estimation based upon published results (including our own; Castrucci et al., 1996, 1998; Nickell et al., 2016; Guerra-Maupome et al., 2019; Mahmoud et al., 2020; Wheat et al., 2020; Díaz et al., 2021) for in vivo innate immunostimulant activity in cattle. Given a medium effect size (following Cohen’s criteria, Cohen, 1988), an α = 0.05, and power = 0.7, the projected samples size to measure a significant effect of SCFP supplementation on gross lung pathology scores (in challenge studies, Mann–Whitney test) was *n* = 12, calculated using the G*Power 3.1 software. The projected sample size to measure an effect of SCFP supplementation on innate immune function compared with untreated animals was *n* = 8 animals/group. Therefore, we chose to enroll *n =* 14 calves in each group to account for possible animal losses. The animals were purchased from a commercial dairy and transported approximately 2 h to the Livestock Infectious Disease Isolation Facility at Iowa State University. Enrollment criteria were based on a satisfactory health assessment upon arrival. Prior to purchasing, adequate passive transfer status for each calf was confirmed on-farm by using a refractometer with a cutoff for enrollment at >5.5 g/dL serum total protein. A total of 28 calves were purchased; however, one animal failed the health assessment at arrival and was rejected from the trial. The calves were blocked by initial body weight and then randomized into two treatment groups: 1) control (*n* =13; 5 males and 8 females); base milk replacer and calf starter; 2) SCFP supplemented (*n* =14; 7 males and 7 females). The SCFP (Diamond V, Cedar Rapids, IA) were fed at a rate of 1 g/d (SmartCare, Soluble SCFP, Diamond V, Cedar Rapids, IA) suspended in milk (0.5 g/feeding) and 5 g/d top dressed onto the calf starter (NutriTek, Insoluble SCFP, Diamond V, Cedar Rapids, IA).

The calves were housed indoors in the environmentally controlled BSL-2Ag facility for the duration of the study. The animals were housed individually on the floor with pine chip bedding. Bedding was picked daily and stripped every third day. Calves were housed in four large rooms. Two rooms housed eight calves each (four SCFP-treated and four controls); one room housed seven calves (four SCFP-treated and three controls); and one room housed four calves (two SCFP-treated and two controls). All calves had ad libitum access to fresh water using an automatic watering system. Calf starter grain was offered in individual plastic troughs. Starter was weighed each morning at approximately 0800 hours and daily consumption was recorded. Leftover starter was discarded, the trough was cleaned, and fresh calf starter was replaced daily (with SCFP top dressed, as appropriate). Calves were initially offered 0.23 kg starter grain, which was increased over time to maintain a ~15% refusal rate. The calf starter was pelleted and free of yeast products and ionophores. The ingredient and nutrient composition of calf starter grain is identical to that reported by Mahmoud et al. (2020) and can be found in Supplementary Table S1.

Calves were fed milk replacer using nipple bottles. Milk replacer was offered twice per day at approximately 0700 and 1800 hours. After 15 min, refusals were fed via stomach tube to ensure the entire contents were consumed. Both consumption and any requirement for tube feeding were recorded. SCFP treatments were mixed into individual bottles (0.5 g/feeding) and shaken to disperse. Milk replacer was custom formulated to contain no yeast or ionophores and was identical to that reported by Mahmoud et al. (2020; Supplementary Table S1). Calves were fed at a rate of 1 kg solids/d at 15.5% w/v.

### BRSV infection

Calves were infected with BRSV at approximately 3 wk of age. Calves were divided into three groups for logistical purposes during experimental infection, with infections starting on days 20, 21, and 22 of the study. The viral infections were staggered by room and groups of calves were infected every 24 h, with seven to eight calves infected/d. Calves were infected with ~10^4^ Median Tissue Culture Infectious Dose (TCID_50_) BRSV strain 375 via nebulizer aerosol inoculation covering the nostrils and mouth as described by McGill et al. (2018). Following infection, room order was followed to eliminate potential cross-contamination between infected and uninfected rooms. To generate the virus stock, virus was freshly isolated from diseased lung and passaged twice on primary bovine turbinate cell as in McGill et al. (2018). Approximately 5 mL of the viral inoculum were delivered via forced-air nebulizer to a mask covering the calf’s mouth and nose.

### *PM* infection

Calves were infected with *PM* on day 6 after BRSV infection (days 26, 27, and 28, depending upon group). Calves were challenged via intratracheal inoculation by insertion of a 16 g needle inserted through three laryngeal rings distal to the pharynx. Calves were challenged with ~10^10^ colony forming units (**CFU**) *PM* strain P1062 type A:3 suspended in 60 mL saline, followed by a 30 mL phosphate buffered saline (**PBS**) to wash the solution into the lungs. The bacterial culture was prepared using an adaptation of previously described methods (Dagleish et al., 2010; Gershwin et al., 2015). Briefly, pure cultures of *PM* were plated on brain–heart infusion (BHI) agar plates 2 d prior to challenge. The morning of the challenge, pure colonies were suspended in 17 mL BHI broth and grown to log-phase in a 37 °C shaking incubator. After ~2 to 3 h, the bacterial cells were pelleted and washed several times with PBS, then resuspended in saline for inoculation. The infection dose administered to each calf was confirmed by quantitative culturing on BHI agar.

### Blood collection

Blood was collected aseptically via the jugular vein immediately prior to infection and on days 6, 7, and 10 after BRSV infection. All samples were collected in the morning at approximately 0600 hours, prior to the first milk feeding of the day. Blood was collected into marble-top serum separator tubes and allowed to clot. Approximately 2 h later, blood was centrifuged, and serum was aliquoted to be stored at −80 °C until later analysis.

### Liver biopsy

A liver biopsy was collected from each calf on day 14 of the trial (approximately 7 d prior to viral infection). Calves were clipped on the right side of their body between the 10th and 12th rib. Liver biopsy sites were disinfected using betadine, 70% ethyl alcohol, and a soft-bristled brush. Around 0.5 mL of a local anesthetic (lidocaine) was administered at each of the three injection sites on each side of the 11th intercostal space, creating two parallel lines. Using a 22-blade scalpel, an incision parallel to the ribs was made between the 11th and 12th rib on a line from the hip to the point of the calf’s shoulder. Immediately following incision, liver tissue was collected using a two-pieced biopsy probe (Jamshidi bone marrow needle) with a fitted trocar. Following collection, samples were expelled from the syringe, rinsed with cold PBS, stored in a microcentrifuge tube, and placed on ice before transporting back to the laboratory. A second liver biopsy was collected at necropsy on day 10 after BRSV infection. Both sample sets were stored in RNAlater (Thermo Fisher Scientific) at −80 °C for future analysis of metabolic, endocrine, and cytokine relative gene expression.

### Performance data

Calf starter and milk replacer intake were recorded daily. Calves were weighed five times during the trial: at arrival, weekly during the feeding period, and immediately prior to euthanasia.

### Clinical illness scoring

Calves were monitored daily for clinical disease by a single trained observer. Scoring was determined by assigning a score of 0 to 3 based on severity of clinical signs including rectal temperature, ocular and nasal discharge, cough, ear position, head tilt, respiratory rate, respiratory effort, and lung auscultation. Calves were scored using the scoring system described in Mahmoud et al. (2020) which has been adapted from Dr. Sheila McGurik’s University of Wisconsin Calf Health Respiratory Scoring Chart (https://www.vetmed.wisc.edu/fapm/svm-dairy-apps/calf-health-scorer-chs/).

### Thoracic ultrasonography

On days 7 and 10 following BRSV infection, thoracic ultrasonography (**TUS**) was performed with an IBEX EVO (E.I. Medical Imaging, Loveland, CO) using the L7HD linear transducer probe (5 to 9 MHz) set to a depth of 8.7 cm for all scans with 70% isopropyl alcohol applied to the areas of interest. Thorax of all calves were clipped within 1 wk prior to the start of challenge to improve image quality. Nine locations on the calf were identified for ultrasonography with five locations on the left side of the calf and four locations on the right side of the calf. Ultrasound locations were based on previously described locations of interest (Rademacher et al., 2013). At each location, a four-second clip was recorded and stored for later image review. Ultrasound clips for each location were scored for presence of pleural defects and depth of consolidation. The scoring system was modified from previous published reports (Rademacher et al., 2013; Timsit et al., 2019; Cuevas-Gómez et al., 2020; Buczinski et al., 2021). Pleural defects were assigned a score of 0 to 3, 0: no comet tails/B-lines observed; 1: 1 to 2 comet tails/B-lines observed; 2: 3 to 5 comet tails/B-lines observed; 3: 6+ comet tails/B-lines observed. Depth of consolidation was also assigned a score of 0 to 3 based on the maximum depth of consolidation seen at each location, 0: no consolidation; 1: less than 2 cm of consolidation; 2: greater than 2 cm but less than 4 cm of consolidation; and 3: greater than 4 cm of consolidation. Once all nine locations for a day were scored, the scores for presence of pleural defects were added for a final pleural defect score and the maximum consolidation depth seen in any of the nine locations on each day was assigned as the final depth of consolidation score. Calves were assigned a final ultrasound score for the day on a scale of 0 to 4, 0: no consolidation present, final pleural defects score of <5; 1: no consolidation present, final pleural defects score >5; 2: final depth of consolidation score of 1; 3: final depth of consolidation score of 2; and 4: final depth of consolidation score of 3.

### Necropsy and pathological evaluation

Calves were humanely euthanized on day 10 post-viral infection by barbiturate overdose. Pathological evaluation was performed similar to previous descriptions (Viuff et al., 2002; Sacco et al., 2012). Lungs were graded on pneumonic consolidation using a previously published scoring system (McGill et al., 2018). The lungs were assigned a score based on the percentage of lung affected by gross pneumonic lesions (0 = free of lesions; 1 = 1% to 5% affected; 2 = 6% to 15% affected; 3 = 16% to 30% affected; 4 = 31% to 50% affected; 5 = >50% affected). Affected and unaffected lung tissues were collected and stored in RNAlater for real-time polymerase chain reaction (**qPCR**) analysis. Liver samples were collected from each calf and stored at −80 °C for qPCR analysis.

Bronchoalveolar lavage (BAL) fluid was collected by introducing 500 mL of sterile, ice-cold saline with antibiotics through the trachea. The fluid was recovered by transferring into sterile collection bottles. Collected BAL samples were filtered through sterile gauze, and duplicate samples were submitted to the Iowa State University Veterinary Diagnostic Laboratory for preparation of cytospins and differential staining (Modified Wrights Stain, Sigma-Aldrich, St. Louis, MO). A blinded clinical pathologist performed differentials with a minimum of 400 cells counter per slide.

### Enzyme-linked immunosorbent assay

The following commercial enzyme-linked immunosorbent assay (**ELISA**) kits were used for analysis of bovine metabolic, endocrine, and immune targets in serum samples: haptoglobin (Bovine Haptoglobin ELISA kit, Immunology Consultants Laboratory, Portland, OR); insulin (Bovine Insulin ELISA kit, Merocodia, Upsala, Sweden); interleukin-6 (IL-6; Bovine IL-6 ELISA kit, Thermo Fisher Scientific). Each ELISA was performed per manufacturer’s instructions, and data were collected on a Thermo Fisher Multiskan FC Microplate Photometer. Serum concentrations of nonesterified fatty acids (**NEFA**) were determined using the reagents provided in the FujiFilm HR Series NEFA-HR (2) assay kit (Fujifilm, Valhalla, NY). The protocol for NEFA in serum was adapted from Johnson and Peters (1993) for manual 96-well microplate analysis. Serum concentrations of beta-hydroxybutyric acid (**BHB**) were determined using the MedTest Pointe β-Hydroxybutyrate Reagent set per manufacturer instructions. Reactions were performed in 3 mL cuvettes and the data were collected on a Bio-Rad SmartSpec 3000 (Bio-Rad Laboratories, Hercules, CA). Serum glucose concentrations were determined using Glucose Autokit (Fujifilm, Valhalla, NY) per the manufacturer microtiter instructions. Serum blood urea nitrogen (**BUN**) was determined using a commercial Urea Nitrogen kit (Teco Diagnostics, Anaheim, CA).

### Real-time PCR

Viral load was determined in nasal swabs and lung tissue collected at necropsy. The ribonucleic acid (RNA) isolation for each sample was performed as described (McGill et al., 2018). Representative lung samples underwent RNA isolation with Trizol reagent (Thermo Fisher Scientific) and nasal swabs were placed in virus free isolation media and isolated according to the manufacturer’s protocol (MagMAX Viral RNA Isolation Kit; Thermo Fisher Scientific). Briefly, paramagnetic beads with an RNA binding surface were added to the samples to bind RNA. The beads/RNA were captured on magnets, and proteins and other contaminants were washed away. The beads were then washed again to remove residual binding solution. RNA was eluted in elution buffer. Viral NS2 gene calculations and qPCR were performed using the Taqman RNA-to-CT 1-step kit (Applied Biosystems) and normalized to the S9 housekeeping gene to correct for differences in the input material as described in Mahmoud et al. (2020; Supplementary Table S2).

Pre- and post-infection liver RNA samples were isolated and complimentary deoxyribonucleic acid (**cDNA**) was prepared as in McGill et al. (2018). The qPCR reactions were performed using SYBR Green Power PCR Mastermix (Thermo Fisher Scientific) and QuantStudio 3 qPCR Machine (Thermo Fisher Scientific) with the following cycling conditions: 2 min at 50 °C, 10 min at 95 °C, 40 cycles of 15 s at 95 °C and 1 min at 60 °C, and a dissociation step (15 s at 95 °C, 1 min at 60 °C, 15 s at 95 °C, 15 s at 60 °C). Relative gene expression was determined using the 2^−ΔΔCt^ method with RPS9 as the reference housekeeping gene. The primer sets for bovine growth hormone receptor (**GHR**), insulin-like growth factor 1 (**IGF-1**), insulin-like growth factor 1 receptor (**IGF-1R**), insulin receptor (**INSR**), Haptoglobin primers were published by Hiss et al. (2004). The primer sets for tumor necrosis factor alpha (TNF-α), interleukin-1 beta (IL-1β), and IL-6 tumor necrosis factor alpha (TNF-α), interleukin-1 beta (IL-1β), and IL-6 are listed in Supplementary Table S2.

### Bacterial recovery

Approximately 1 g of lung tissue was collected from seven predesignated areas of the lung during necropsy. Instruments and surfaces were decontaminated between each necropsy to minimize cross-contamination. Samples were washed in tap water to minimize environmental contamination and then frozen at −80 °C. To isolate *PM*, lung samples were thawed, homogenized using an Omni International Micro hand-held Homogenizer (Omni International, Kennesaw, GA), and cultured on 5% Columbia sheep blood and Mackay agar plates incubated at 35 °C for 24 h. To avoid contamination, samples were processed in a Class II biosafety cabinet and individual autoclaved homogenizer probes were used. Plates were observed for mucoid appearance for *PM* and colony counts were enumerated. Plates were incubated for an additional 24 h to observe any additional growth. The remaining tissue homogenate was diluted 1:10 in BHI broth and incubated on a shaker for 22 h. Bacterial DNA was extracted using heat extraction method (Ewers et al., 2006) to confirm bacterial identity with qPCR. A cycle threshold (**CT**) value of less than 35 was considered positive identification. Select samples with secondary colony growth from each group were sent to the Iowa State University Veterinary Diagnostic Laboratory for testing with the Bovine Respiratory Viral and Bacterial PCR panel and MALDI-TOF to confirm the culture identity.

### Statistics

Graphs and statistical analyses were performed using GraphPad Prism v9.0.2 (Graphpad Software, Inc.). Experiments were analyzed as a randomized complete block design. Individual calves were considered the experimental unit. For data in which assessments were performed on multiple days, results were analyzed by a linear mixed-effects model, fit using restricted maximum likelihood with the Geisser–Greenhouse correction, followed by Sidak’s test for multiple comparisons. The model used the fixed effects of time, treatment (control vs. SCFP diet), and treatment × time interaction. Calf was considered a random effect. Single measurement data were analyzed by unpaired *t*-test or Mann–Whitney test, as indicated in the respective figure legends. Data are reported as least squares means ± SEM. Differences of *P* ≤ 0.05 were considered significant, and tendencies were declared at 0.05 < *P* ≤ 0.10.

## Results

### Calf enrollment and losses

Calves were screened for serum total protein levels as a condition for trial enrollment. The mean serum total protein for calves assigned to the control group was 5.95 ± 0.06 g/dL and was 5.90 ± 0.09 g/dL for the SCFP group. Serum total protein values at enrollment did not differ between groups (*P* = 0.48). A total of 28 animals were purchased for the trial. One calf in the control group failed the initial enrollment health screening and was rejected from the trial. One calf from the SCFP-treated group was euthanized on day 19 of the study (prior to the infection period). The calf displayed fever, lethargy, and inappetence. The calf was treated with a two-dose course of ceftiofur, flunixin meglumine, and supportive care. The animal failed to recover and was euthanized for humane reasons. At necropsy, the calf was diagnosed with nephritis which was likely secondary to previous bacterial sepsis.

### Performance

**Figure 1.**
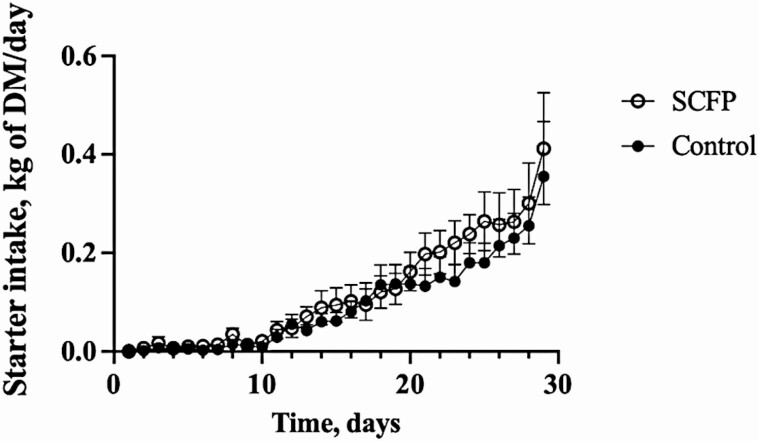
Effects of SCFP supplementation and viral–bacterial coinfection on calf starter intake on days 1 to 30. Calf starter intake was monitored daily in control (*n* = 13) and SCFP-treated calves (1 g/d SmartCare, 5 g/d NutriTek, *n* = 14). One SCFP calf was removed from the study on day 19. The data for this calf are included until the day that the animal was removed. All remaining calves (*n* = 13 controls, *n* = 13 SCFP-treated) were challenged via aerosol inoculation with bovine respiratory syncytial viral strain 375 on days 20 to 22. Calves were then challenged via intratracheal inoculation with *PM* on days 26 to 28. One control calf was euthanized after reaching clinical endpoint on day 9 post-viral infection (day 29). The data for this calf are included until the animal was removed. Data are presented as means ± SEM.

There were no differences between the control and SCFP-fed groups in initial (*P =* 0.65) or final (*P =* 0.53) body weight (Table 1). Mean starter intake did not differ (*P =* 0.47) between the treatment groups (0.095 and 0.118 kg/d for the control and SCFP calves, respectively). However, consistent with our previous study (Mahmoud et al., 2020), there was a time effect, with increased starter intake as the animals aged (*P <* 0.0001; Table 1; Figure 1). There was no difference in average daily gain between groups (*P =* 0.89).

**Table 1.** Effects of treatment on performance measurements in calves supplemented with or without SCFP for 32 d

Parameter	Control	SCFP^1^	SEM	*P*-value		
				Treatment	Time	Treatment × time interaction
Initial BW^2^, kg	40.6	39.6	1.93	0.65		
Final BW, kg	55.6	54.2	1.97	0.53		
Starter intake, kg/d	0.0950	0.118	0.0210	0.47	<0.0001	0.99
ADG^3^, kg/d	0.516	0.522	0.0370	0.89		

^1^SCFP, calves fed *S. cerevisiae* fermentation products, 1 g/d SmartCare suspended in milk and 5 g/d NutriTek on calf starter.

^2^BW, body weight.

^3^ADG, average daily gain.

### Virus and bacteria coinfection

**Figure 2.**
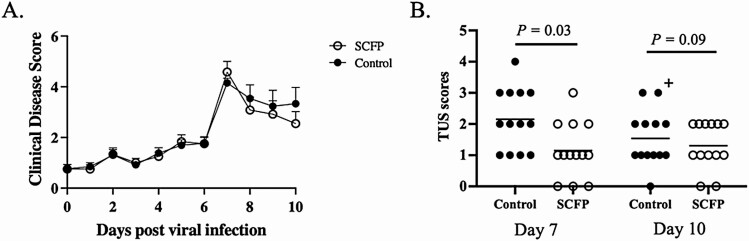
Clinical disease and thoracic ultrasonography (TUS) scores following bovine respiratory syncytial virus (BRSV)-*PM* coinfection in control and SCFP-treated calves. Following BRSV-*PM* coinfection, clinical signs were monitored by a single trained observer and scores were assigned daily using the system described in Materials and Methods. Parameters include rectal temperature, expiration effort, respiration rate, ocular and/or nasal discharge, cough, and lung auscultation. *n* = 13 SCFP-treated calves. *n* = 13 control calves. One control calf reached a humane endpoint and was euthanized on day 9 post-viral infection (day 29) (A). Lung disease progression was evaluated using TUS on days 7 and 10 post-viral infection. Scores were assigned as described in Materials and Methods. On day 7 after viral infection, SCFP-treated calves (1 g/d SmartCare, 5 g/d NutriTek) had lower TUS scores (*P =* 0.03) than control calves (*n* = 13 controls; *n* = 13 SCFP-treated). On day 10 after viral infection, both groups experienced lower scores, with SCFP-treated calves experiencing tendency to lower scores (*P =* 0.09) than the controls (*n* = 13 controls; *n* = 13 SCFP-treated). One calf was removed from the study on day 9 after infection. TUS data were collected from this animal prior to euthanasia and are included in the day 10 results (B). Plus symbol indicates the score of the animal analyzed on day 9. Data are presented as means ± SEM.

The BRSV-*PM* coinfection resulted in respiratory disease symptoms that included elevated temperatures, coughing, and increased respiratory effort. Clinical signs became apparent on days 4 to 6 following the initial viral infection and increased sharply on day 7, 24 h following the secondary bacterial infection (Figure 2A). Following peak clinical disease on day 7 after infection, clinical scores slowly declined but remained elevated until necropsy on day 10 after BRSV infection. One control calf reached a humane endpoint on day 9 after BRSV infection (48 h after *PM* inoculation) and was euthanized. The humane endpoint was defined as a prolonged increased in body temperature (>40.5 °C for more than 48 h), inappetence for more than two feeding periods and respiratory effort scores of 3 for more than 48 h. Prior to euthanasia, the calf underwent TUS scoring. Postmortem, the calf was necropsied for gross pathology evaluation and collection of lung tissue samples but was not subjected to BAL collection for cytology analysis. The remaining calves survived until day 10 after BRSV infection. We observed no treatment (*P =* 0.69) or treatment by time effects (*P =* 0.83) in clinical disease scores between control and SCFP-fed calves; however, we observed an effect of time (*P <*0.0001). Given the known poor sensitivity of clinical observations, we also monitored disease progression by TUS (Figure 2B). On day 7 post-BRSV infection (24 h after *PM* inoculation), calves in both groups developed elevated TUS scores; however, SCFP-fed calves developed lower TUS scores (*P =* 0.03) compared with control calves. By day 10 after infection, TUS scores declined in both groups, indicating that the animals were resolving the infection. However, SCFP-fed animals still tended to have lower TUS scores (*P* = 0.09) compared with untreated controls.

**Figure 3.**
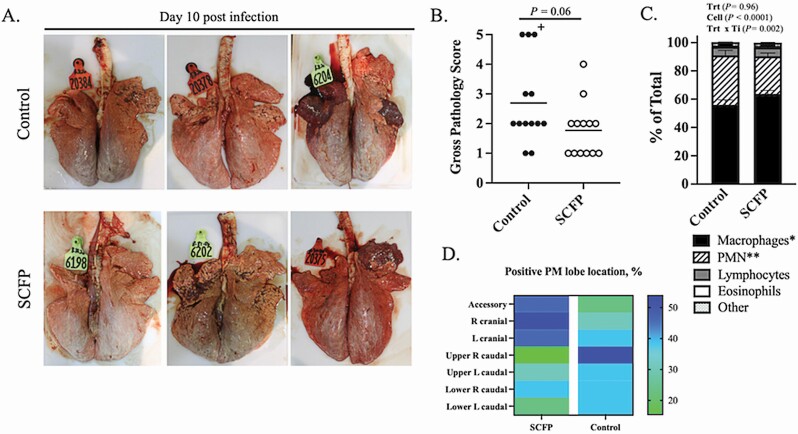
Calves receiving SCFP supplementation tend to develop less bovine respiratory syncytial virus-*PM* induced lung pathology and less neutrophil infiltration into the airways. On day 10 post-viral infection, the calves were humanely euthanized and necropsied. The extent of gross pneumonic consolidation was evaluated based upon the percentage of lung affected (0 = free of lesions; 1 = 1% to 5% affected; 2 = 6% to 15% affected; 3 = 16% to 30% affected; 4 = 31% to 50% affected; 5 = >50% affected). Representative gross lung lesions from three individual calves in the control group (top) or SCFP-treated group (1 g/d SmartCare, 5 g/d NutriTek; bottom) (A). Aggregate gross pathology results from all calves (B). Plus symbol indicates the score of the control calf that was euthanized on day 9. BAL samples were collected from all calves (SCFP-treated, *n* = 13; and controls, *n* = 12) on day 10 post-viral infection (C). Cytospin preparations were made and differentially stained with Modified Wrights Stain. Relative numbers of neutrophils, macrophages, lymphocytes, eosinophils, and other cell types were determined by microscopy. The relative numbers of macrophages (*P = 0.07) and neutrophils (**P = 0.04) present in the lung airways differed between SCFP and control calves. Data are presented as means ± SEM. The lung location and percentage of lung lobes positive for *PM* bacterial recovery in each treatment group (D).

On day 10 post-viral infection, all surviving calves were humanely euthanized, and a necropsy was performed. The lungs were evaluated and assigned a gross pathology score based on the area of consolidation as previously described (McGill et al., 2018, 2019). Representative images of lungs from three control (top panel) and three SCFP-treated (bottom panel) animals are depicted in Figure 3A. Calves in both groups developed bilateral, primarily coalescing pneumonic consolidation with beginning cranial multifocal pneumonic consolidation (McGill et al., 2018, 2019). The SCFP calves tended (*P =* 0.06) to develop fewer gross lung lesions and had lower lung pathology scores compared with control calves (Figure 3B). Recruitment of neutrophils to the airways during respiratory infection is commonly correlated with lung inflammation and disease severity. Cytology was performed on the BAL fluid to determine the ratios of cell types in the lung airways following infection (Figure 3C). Differential counts revealed fewer PMNs (*P =* 0.04) and a higher frequency of macrophages (*P =* 0.07) present in the airways of SCFP-treated calves compared with controls, corresponding to the overall reduced gross lung pathology scores in treated calves.

**Figure 4.**
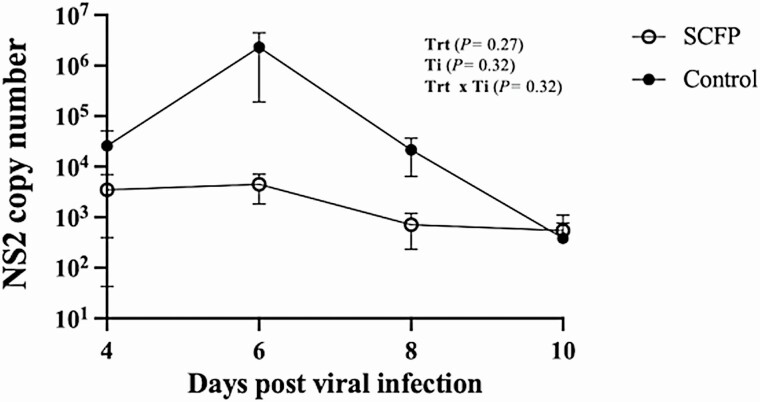
Calves supplemented with SCFP tended to have reduced viral shedding compared with control calves. Nasal swabs were collected from all animals on days 0, 2, 4, 6, and 8 post-viral infection (SCFP-treated, 1 g/d SmartCare, 5 g/d NutriTek), *n* = 13; and controls, *n* = 13) and from all remaining animals on day 10 post-viral infection (SCFP-treated, 1 g/d SmartCare, 5 g/d NutriTek), *n* = 13; and controls, *n* = 12). Swabs were snap-frozen at −80 °C until later analysis. Virus shedding was quantified by qPCR for the bovine respiratory syncytial virus NS2 gene. NS2 mRNA was undetectable in all animals on days 0 and 2 post-viral infection. Data are presented as means ± SEM.

Viral shedding in nasal swabs was quantified using qPCR to detect the BRSV NS2 gene. Virus was detectable in nasal swabs beginning on day 4 after BRSV infection and sustained in some animals until day 10 after infection (Table 2; Figure 4). Virus was detected with similar frequency, and the quantity of virus being shed did not differ between groups (*P =* 0.33). Viral lung load was also measured by qPCR on lung tissue on day 10 after infection (Table 2). Virus was detected in 4/13 control calves and 3/13 SCFP-fed calves, and the viral load did not differ between groups (*P* = 0.48). Cultivation of *PM* from seven predetermined lung lobe locations was performed to quantify bacterial burden. There was not a difference between groups in the frequency of lung lobes positive for *PM* recovery on day 10 after infection (11/13 control calves vs. 11/13 SCFP-fed calves, *P* = 0.90). The mean bacterial load of *PM* recovered from the lungs of control calves was 3.94 × 10^7^ ± 3.63 × 10^7^, and from SCFP calves was 2.07 × 10^5^ ± 1.89 × 10^5^ and did not differ between treatment groups (*P =* 0.34). However, we observed a notable numerical difference in the lobe locations that were positive for *PM* recovery. Calves treated with SCFP had *PM* recovered more frequently from the cranial lung lobes (19/39 SCFP cranial lobes and 12/39 control cranial lobes), whereas the control calves that had *PM* recovered more commonly in the caudal lobes (14/52 SCFP caudal lobes and 22/52 control caudal lobes; Figure 3D).

**Table 2.** Detection of bovine respiratory syncytial virus (BRSV) by virus isolation and qPCR for the BRSV NS2 gene in nasal swabs and lungs after BRSV-*PM* coinfection of calves supplemented with or without SCFP for 32 d

		Nasal swabs							Lung^1^	
		Day 0	Day 2	Day 4	Day 6	Day 8	Day 10	SEM	Day 9 or 10	SEM
Control	Virus isolation	0/13	0/13	4/13	13/13	4/13	1/12		4/13	
	NS gene copies/10^4^ RPS9 copies^2^	ND^3^	ND	25,800	2,313,000	21,400	384	2,120,000	307	306
SCFP^4^	Virus isolation	0/13	0/13	3/13	12/13	3/13	1/13		3/13	
	NS gene copies/10^4^ RPS9 copies	ND	ND	3,490	4,480	715	549	3,450	32	32

^1^These data include the results from the calf in the control group that was euthanized on day 8 post-infection for humane reasons. The lung tissue from this animal was positive for BRSV.

^2^Nasal swabs: treatment effect *P =* 0.33; time effect *P =* 0.43; treatment × time interaction *P =* 0.44. Lung tissue: treatment effect *P =* 0.48.

^3^ND, not detected.

^4^SCFP, calves fed *S. cerevisiae* fermentation products, 1 g/d SmartCare suspended in milk and 5 g/d NutriTek on calf starter.

### Metabolic responses to viral–bacterial coinfection

**Figure 5.**
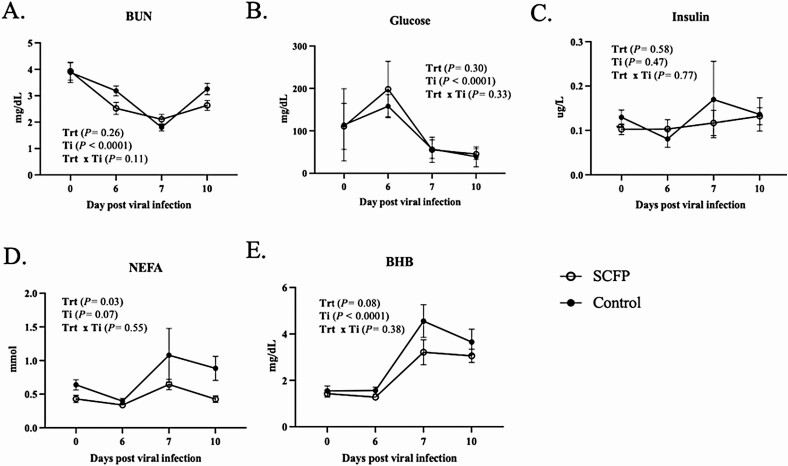
Serum metabolic responses to viral–bacterial coinfection and SCFP supplementation. Serum was collected on days 0, 6, 7, and 10 post-viral infection and analyzed using commercial (A) BUN, (B) glucose, (C) insulin, (D) NEFA, and (E) BHB ELISA kits. SCFP-treated (1 g/d SmartCare, 5 g/d NutriTek), *n* = 13; and controls, *n* = 13. One control calf was removed from the study on day 9 post-viral infection and is not included in the day 10 results. Data are presented as means ± SEM.

Blood serum metabolites were measured on days 0, 6, 7, and 10 post-viral infection to determine the impact of BRDC and SCFP supplementation on metabolic responses (Figure 5). Serum BUN concentrations declined following BRSV infection to a nadir on day 7, which was 24 h following bacterial infection (Figure 5A). Each group experienced changes to BUN concentrations over time (*P <* 0.0001), but we observed no treatment effects on BUN concentrations (*P* = 0.26). Serum glucose concentrations did not differ between treatment groups (*P =* 0.30) but changed over time (*P <* 0.0001), decreasing sharply on day 7 following the secondary bacterial infection (Figure 5B). Serum insulin concentrations did not vary over the infection period (*P* = 0.58); nor were there differences between treatment groups (*P =* 0.47; Figure 5C). We noted an SCFP effect (*P* = 0.03) on serum NEFA concentrations resulting in lower concentrations for the SCFP-treated calves (Figure 5D). There was a tendency for NEFA concentrations to differ over time (*P =* 0.07), with a marked increase in NEFA concentrations in both groups between days 6 and 7 and a decline by day 10 after infection. Serum BHB concentrations changed over time (*P <* 0.0001), with a sharp increase on day 7 in both groups following bacterial coinfection (Figure 5E). The control group experienced a greater increase in BHB from days 6 to 7 and tended to be elevated compared with the SCFP-treated group (*P =* 0.08). Concentrations of BHB decreased slightly from days 7 to 10 but remained elevated through day 10 after virus infection.

### Liver endocrine response to viral–bacterial coinfection

Liver biopsies collected pre- and post-viral infection were evaluated by qPCR for gene expression of several endocrine factors (Table 3). The expression of IGF-1 was similar between treatment groups (*P =* 0.94), but expression of IGF-1 increased (*P =* 0.02) over time. Expression of IGF-1R did not differ between treatment groups (*P =* 0.97) or change over time (*P =* 0.52). Expression of INSR did not differ in response to SCFP treatment (*P =* 0.39) but increased over time (*P* < 0.0001). Expression of GHR did not differ over time (*P =* 0.22) or in response to SCFP treatment (*P* = 0.39).

**Table 3.** Effects of SCFP supplementation on gene expression in liver biopsies collected before and after bovine respiratory syncytial virus (BRSV)-*PM* coinfection

Primer	Control ΔΔCT^1^ Pre-inf^2^	Control ΔΔCT^1^ Post-inf^3^	SCFP^4^ ΔΔCT^1^ Pre-inf	SCFP^4^ ΔΔCT^1^ Post-inf	SEM	*P*-value		
						Treatment	Time	Treatment × time interaction
IGF-1^5^	9.17 E-9	−0.679	0.515	−0.706	0.531	0.94	0.02	0.87
IGF-1R^6^	6.67 E-9	0.386	0.579	0.180	0.439	0.97	0.52	0.38
INSR^7^	0.00	−0.799	−0.209	−0.780	0.145	0.39	<0.0001	0.31
GHR^8^	−8.33 E-9	−0.272	0.325	0.107	0.339	0.22	0.39	0.55
Haptoglobin	−8.33 E-5	−1.587	−0.729	−2.61	0.798	0.16	0.01	0.83
TNF-α	2.84 E-7	−0.937	−0.894	0.521	0.972	0.70	0.66	0.04
IL-6	5.54 E-8	0.196	−0.110	3.09	1.67	0.26	0.09	0.13

^1^ΔΔCT, delta-delta CT, calculated using the 2^−ΔΔCt^ method with RPS9 as the reference housekeeping gene.

^2^Pre-infection liver biopsies were collected 7 d prior to BRSV-*PM* coinfection.

^3^Post-infection liver samples were collected on day 10 after BRSV-*PM* coinfection.

^4^SCFP, calves fed *S. cerevisiae* fermentation products, 1 g/d SmartCare suspended in milk and 5 g/d NutriTek on calf starter.

^5^IGF-1, insulin-like growth factor 1.

^6^IGF-1R, insulin-like growth factor 1 receptor.

^7^INSR, insulin receptor.

^8^GHR, growth hormone receptor.

### Acute-phase response in liver and serum

**Figure 6.**
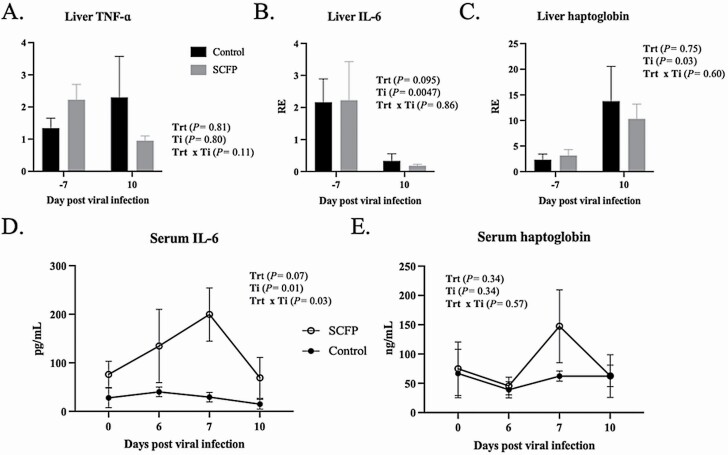
Serum and liver TNF-α, IL-6, and haptoglobin responses in SCFP-treated calves compared with controls following viral–bacterial coinfection. Liver biopsies were collected 7 d prior to bovine respiratory syncytial virus infection and again at necropsy on day 10 after viral infection. RNA was isolated from liver samples and analyzed by qPCR for expression of (A) TNF-α, (B) IL-6, and (C) haptoglobin. Serum samples were collected on days 0, 6, 7, and 10 post-viral infection and analyzed using commercial ELISA kit for concentrations of (D) IL-6 and (E) haptoglobin. SCFP-treated (1 g/d SmartCare, 5 g/d NutriTek), *n* = 13; and controls, *n* = 13. One control calf was removed from the study on day 9 post-viral infection and is not included in the day 10 results. Data are presented as means ± SEM.

We evaluated the effect of SCFP supplementation on the acute-phase response induced by viral–bacterial coinfection by monitoring serum proinflammatory markers on days 0, 6, 7, and 10 post-viral infection. Liver samples were collected approximately 7 d prior to infection and then on day 10 post-infection and evaluated for gene expression of the inflammatory cytokines IL-6 and TNF-α, and the acute-phase protein haptoglobin. No differences (*P >* 0.05) were observed in expression of any of the target genes between SCFP and control calves either prior to, or following, infection. Expression of liver TNF-α did not vary with time (*P* = 0.70), but we noted a treatment × time interaction (*P =* 0.04; Table 3; Figure 6A) suggesting that SCFP-treated calves tended to downregulate liver expression, while control calves tended to upregulate expression. Expression of liver IL-6 tended to be downregulated in both treatment groups by day 10 after viral–bacterial coinfection (Table 3; Figure 6B). In contrast, both groups upregulated expression of haptoglobin in the liver on day 10 after infection (*P =* 0.01; Table 3; Figure 6C).

The serum concentrations of TNF-α, IL-6, and haptoglobin were also quantified on days 0, 6, 7, and 10 after BRSV infection. Serum TNF-α concentrations were below the limit of detection (15 pg/mL) for all timepoints (data not shown). We noted a treatment × time interaction for serum IL-6 concentrations (*P* = 0.03), such that concentrations were increased in SCFP-treated calves on day 7 after viral–bacterial coinfection compared with control calves (*P* = 0.03). By day 10 after infection, serum IL-6 concentrations in SCFP-fed calves declined and were similar to baseline levels (Figure 6D). Serum haptoglobin concentrations increased in some individual animals on days 7 and 10 after infection. However, as a group, concentrations of haptoglobin did not differ over time (*P =* 0.34) or in response to SCFP treatment (*P* = 0.34; Figure 6E).

## Discussion

Respiratory disease carries a high economic burden in all aspects of the cattle industry; for the United States feedlot industry, respiratory disease costs approximately $3 billion annually (USDA, 2010, 2012; Smith et al., 2020). We recently reported that SCFP supplementation modulates both systemic and mucosal immune function, leading to increased resistance to experimental BRSV infection in preweaned calves (Mahmoud et al., 2020). A combination of pathogenic and commensal organisms often results in BRDC. In our previous study, we observed that SCFP-fed calves naturally developed fewer secondary bacterial infections in the day following BRSV infection compared with control calves (Mahmoud et al., 2020). Therefore, in the present trial, we directly addressed the susceptibility of SCFP-fed calves to BRDC using an experimental coinfection model of BRSV with *PM*. Our results suggest a similar beneficial effect of SCFP supplementation on the outcome of a BRDC coinfection model in terms of disease resistance. Although we did not observe differences in clinical signs between treatment groups, SCFP-fed calves tended to be more resistant to the lung damage caused by viral–bacterial coinfection as indicated by TUS scoring and evaluation of gross lung lesions at necropsy. Neutrophil infiltration into the BAL is correlated with BRDC severity (Hägglund et al., 2017). In agreement with the results of our gross lung scores, calves fed SCFP also had fewer neutrophils infiltrating the lung airways compared with control calves. It is well established that BRDC during neonatal development has long-term adverse impacts on performance, and this effect is at least partially attributed to permanent changes in lung capacity following severe respiratory infections (Buczinski et al., 2021). Thus, the capacity for SCFP treatment to reduce lung consolidation during an infection has potential long-term implications for bovine health.

In this trial, we did not observe effects of SCFP supplementation on viral or bacterial lung loads. Interestingly, however, we observed a difference in the distribution of the bacterial pathogen in the lungs of SCFP-fed calves compared with controls. There are many commensal bacterium residing in the nasopharyngeal cavity of cattle including *PM*, but this organism can become pathogenic during stressful events or in the context of a viral infection (Dabo et al., 2007). Pneumonia caused by *PM* typically presents as suppurative bronchopneumonia and is commonly seen in young dairy calves with BRDC (Panciera and Confer, 2010). Infection with BRSV enhances *PM* adherence in the epithelial cells of the lower respiratory tract bronchus (Sudaryatma et al., 2020). Animals affected by BRSV are expected to have a range of cranioventral lesions and caudodorsal inflation-associated lesions (Sacco et al., 2012). Because BRSV enhances *PM* adherence, pathogenic bacterial colonization may follow the viral invasion in the lower respiratory tract (Sudaryatma et al., 2020). Here, we noted that the majority of *PM* recovered from SCFP calves was constrained to the cranioventral lobes, while more *PM* recovered from control calves had spread to the caudodorsal lobes. This difference in bacterial distribution in the lungs may have implications in controlling and clearing the bacterial pathogen. Coupling this data with the gross pathology scores, calves receiving no supplement tended to have more severe and more disseminated lung infection than animals receiving the SCFP supplement. The localized nature of the infection in the SCFP-treated calves may have implications for improved recovery efficiency in the lungs.

A possible explanation for the increased resistance to BRDC in SCFP-treated calves is the apparent immunomodulatory effects of SCFP supplementation on calf immune function. Yeast and yeast products have known effects on immune function in cattle (Burdick Sanchez et al., 2021). Cattle supplemented orally with live yeast have higher numbers of circulating monocytes and neutrophils compared with controls following a coinfection with bovine herpesvirus-1 and *M. haemolytica* (Kayser et al., 2019). Feedlot steers receiving a yeast-derived product during preconditioning had lower incidence of natural BRDC outbreaks (Silva et al., 2018). In our previous publication, we noted that peripheral blood mononuclear cells from SCFP-fed calves produced more TNF-α, IL-1β, and IL-6 in response to innate immune agonists such as LPS and Pam3CSK4, a Toll-Like Receptor 2 agonist (Mahmoud et al., 2020). Consistent with this observation, SCFP-treated calves in the present study had higher IL-6 serum concentrations than control calves following BRSV and *PM* coinfection, suggesting a more robust innate immune response. A recent study examining the effects of SCFP supplementation on LPS challenge in calves made a similar observation, finding that calves receiving the SCFP supplement mounted an increased IL-6 response compared with control calves (Klopp et al., 2020). Interestingly, we did not observe the same significance in the serum haptoglobin response, nor in serum TNF-α. Previous work with SCFP supplementation in piglets receiving an *E. coli* challenge has reported increased concentrations of serum TNF-α in treated piglets compared with controls (Kiarie et al., 2012). Klopp et al. (2020) also noted an effect of SCFP treatment on serum TNF-α in response to LPS challenge in their calf model. It is not clear why we were unable to detect TNF-α in the serum following viral–bacterial coinfection. However, we speculate that we may have missed the sampling window for TNF-α, given the rapid kinetics of the TNF-α response. Klopp et al. (2020) measured a rise in serum TNF-α at 1 to 2 h after LPS challenge, which resolved by 4 h after LPS treatment. Although the two types of challenge models differ, it seems likely that serum TNF-α may have spiked very quickly after secondary *PM* challenge and returned to baseline before the next sampling period. Serum haptoglobin is a common marker of the acute-phase response that rises in calves with BRDC (Godson et al., 1996). Serum haptoglobin concentrations positively correlate with serum IL-6 responses (Godson et al., 1996). In our trial, the haptoglobin response was not statistically significant, with some animals experiencing a rise in haptoglobin after infection, and others not. A recent post hoc study of feedlot steers challenged with bovine herpesvirus-1 and *M. haemolytica* revealed that haptoglobin responses were only detectable in 9/18 challenged steers, despite similar body temperatures and clinical disease signs (Wottlin et al., 2020). Interestingly, both the pre- and post-infection feeding behavior patterns were more impacted in haptoglobin-responsive animals compared with the nonresponsive animals, suggesting lower haptoglobin responses may correlate with disease resiliency in feedlot cattle. Together, our results suggest that SCFP supplementation may increase systemic IL-6 responses in calves with bovine respiratory disease, but may not impact other markers of the acute-phase response.

Mounting an effective immune response during infection and inflammation is energetically costly and can affect bovine performance. Calves enrolled in this study saw an increase in serum glucose between days 0 and 6 post-viral infection. The animals had only mild clinical symptoms in the first 6 d following BRSV infection. After receiving the bacterial challenge on day 6 post-infection, the calves experienced a decrease in serum glucose concentrations. Immune cells rely heavily on glucose for energy, and this decline in glucose is consistent with a hypermetabolic state known to occur during disease (Marcato et al., 2018). Montgomery et al. (2009) observed lower glucose levels in feedlot heifers treated for BRDC, and similar observations have been made in field studies of calves diagnosed with natural BRDC compared with healthy calves (Helena et al., 2015). In the present study, coinciding with the decline in glucose on day 7, there was an increase in both NEFA and BHB concentrations. An increase in NEFA and BHB indicates mobilization of fat resources for energy. In veal calves, increases in NEFA and BHB are observed following stressful events such as long duration transport (Marcato et al., 2018). In our trial, calves treated with SCFP had lower concentrations of NEFA and BHB during the coinfection period, suggesting that treated calves may have had less need to mobilize triacylglycerides for energy. This trend could correspond to the overall milder disease observed in the SCFP calves but is interesting considering the more robust immune response, and presumably a higher energetic demand, observed in the SCFP-treated calves compared with controls. Considering the nutrient profiles of SmartCare (27% protein, 0.5% fat, and 1% fiber) and NutriTek (18% protein, 1.2% fat, and 20% fiber) and their feeding rates (1 and 5 g/d, for SmartCare and NutriTek, respectively) caloric contribution from the supplements to daily energy intake would be very minimal relative to that from milk and starter grains. Thus, these effects are difficult to attribute to differences in direct energy intake from the supplement and instead seem to correspond to SCFP effects on metabolic partitioning.

Calves in this study demonstrated changes in BUN over the infection period. We did not observe an effect of SCFP supplementation on BUN concentrations compared with control animals. Our results differ from the study by Alugongo et al. (2017) that evaluated the impact of SCFP supplementation on juvenile Holstein calves. In this study, they observed a tendency for lower BUN levels in SCFP supplemented calves and speculated that this reduction could be the result of the presence of a more robust ruminal population and thus an increase in ruminal nitrogen requirements (Alugongo et al., 2017). Most calves in their study experienced gastrointestinal clinical disease, but animals receiving SCFP had an overall lower incidence and severity of diarrhea, which may have had some impact on serum BUN concentrations.

In summary, calves receiving SCFP treatment mounted an enhanced systemic inflammatory response and developed less lung damage following experimental BRSV-*PM* coinfection. Despite the increased immune response mounted by the SCFP calves, the animals demonstrated metabolic prioritization as evidenced by lower concentrations of serum BHB and NEFA in the post-infection period. Our results suggest that supplementation with SCFP allowed calves to mount a robust immune response and regulate the severity of a viral–bacterial respiratory infection without detrimental metabolic changes. Therefore, these results support further research into the use of SCFP supplementation in growing calves as a nonantibiotic strategy to reduce the incidence and severity of respiratory disease.

## Supplementary Material

skab300_suppl_Supplementary_Tables_S1-S2

## Abbreviations

BAL: bronchoalveolar lavage
BHB: beta-hydroxybutyric acid
BHI: brain–heart infusion
BRDC: bovine respiratory disease complex
BRSV: bovine respiratory syncytial virus
BUN: blood urea nitrogen
cDNA: complimentary deoxyribonucleic acid
CFU: colony forming unit
CT: cycle threshold
ELISA: enzyme-linked immunosorbent assay
GHR: growth hormone receptor
IGF-1: insulin-like growth factor 1
IGF-1R: insulin-like growth factor 1 receptor
IL: interleukin
INSR: insulin receptor
LPS: lipopolysaccharide
mRNA: messenger ribonucleic acid
NEFA: nonesterified fatty acids
PBS: phosphate buffered saline
PCR: polymerase chain reaction
PM: *Pasteurella multocida*
RNA: ribonucleic acid
SCFP: *Saccharomyces cerevisiae* fermentation products
TCID: tissue culture infectious dose
TNF-α: tumor necrosis factor alpha
TUS: thoracic ultrasonography
