# Supplementary material for: Feeding Saccharomyces cerevisiae fermentation products lessens the severity of a viral–bacterial coinfection in preweaned calves
Source: J Anim Sci. 2021 Oct 21;99(11):skab300. doi: 10.1093/jas/skab300 (PMC8599294; doi:10.1093/jas/skab300)
Supplement: skab300_suppl_Supplementary_Tables_S1-S2 [file skab300_suppl_supplementary_tables_s1-s2.docx]

Supplemental Data Table 1: Milk replacer and calf starer formulations for calves supplemented with or without SCFP products for 32 d^1^

| **Ingredient^2^, % DM** | **Milk replacer^3^** | **Starter^4^** |  |  |  |
| --- | --- | --- | --- | --- | --- |
|  |  |  |  |  |  |
| ***Nutrient composition, DM basis*** |  |  |  |  |  |
| **Dry mater, %** | 97 | 94 |  |  |  |
| **Crude protein, 5** | 22 | 19 |  |  |  |
| **Fat, %** | 20 | 2.8 |  |  |  |
| **ADF, %** | 0.15 | 9 |  |  |  |
| **Calcium, %** | 0.78 | 1.7 |  |  |  |
| **Phosphorus, %** | 0.632 | 0.58 |  |  |  |
| **Vitamin A, added IU/kg** | 44,000 | 36,300 |  |  |  |
| **Vitamin D, added IU/kg** | 16,500 | 11,000 |  |  |  |
| **Vitamin E, added IU/kg** | 294 | 330 |  |  |  |

^1^Calves fed SCFP received 1 g/d SmartCare in milk and 5 g/d NutriTek top-dressed on the starter for the duration of the study.

^2^ADF, acid detergent fiber; DM, dry matter

^3^Milk products, Chilton, WI.

^4^Effingham Equity, Effingham, IL.

Supplemental Data Table 2: Primer sequences used in lung and liver gene expression analysis

| **Primer** | **Sequence (5’ to 3’)** | **Citation** |  |  |  |
| --- | --- | --- | --- | --- | --- |
|  |  |  |  |  |  |
| **RPS9, F^1^** | GTGAACATCCCGTCCTTCAT | McGill et al., 2018 |  |  |  |
| **RPS9, R^1^** | TCTTGGCGTTCTTCCTCTTC |  |  |  |  |
| **RPS9, probe^1^** | 56-FAM/AAGTCGATG/ZEN/TGCTTCTGCGAGTCC/3IABkFQ |  |  |  |  |
| **NS2, F^1^** | GAACGACAGGCCACATTTA |  |  |  |  |
| **NS2, R^1^** | AGGCATTGGAAATGTACCATA |  |  |  |  |
| **NS2, probe^1^** | 56-FAM/TGAAGCTAT/ZEN/TGCATAAAGTGGGTAGCACA/3IABkFQ |  |  |  |  |
| **GHR, F^2^** | CCAGTTTCCATGGTTCTTAATTAT | Schäff et al., 2016 |  |  |  |
| **GHR, R^2^** | TTCCTTTAATCTTTGGAACTGG |  |  |  |  |
| **IGF-1, F^2^** | TCGCATCTCTTCTATCTGGCCCTGT |  |  |  |  |
| **IGF-1, R^2^** | GCAGTACATCTCCAGCCTCCTCAGA |  |  |  |  |
| **IGF-1R, F^2^** | TTAAAATGGCCAGAACCTGAG |  |  |  |  |
| **IGF-1R, R^2^** | ATTATAACCAAGCCTCCCAC |  |  |  |  |
| **INSR, F^2^** | TCCTCAAGGAGCTGGAGGAGT |  |  |  |  |
| **INSR, R^2^** | GCTGCTGTCACATTCCCCA |  |  |  |  |
| **Haptoglobin, F^2^** | GTCTCCCAGCATAACCTCATCTC | Hiss et al., 2004 |  |  |  |
| **Haptoglobin, R^2^** | AACCACCTTCTCCACCTCTACAA |  |  |  |  |
| **TNF-α, F^2^** | CGGGGTAATCGGCCCCCAGA | Sacco et al., 2012 |  |  |  |
| **TNF-α, R^2^** | GGCAGCCTTGGCCCCTGAAG |  |  |  |  |
| **IL-1β, F^2^** | ATGGGTGTTTCTGCATGAG |  |  |  |  |
| **IL-1β, R^2^** | AAGGCCACAGGAATCTTG |  |  |  |  |
| **IL-6, F^2^** | CTGAAGCAAAAGATCGCAGATCTA |  |  |  |  |
| **IL-6, R^2^** | CTCGTTTGAAGACTGCATCTTCTC |  |  |  |  |

^1^SYBR Green PCR chemistry was performed.

^2^TaqMan PCR chemistry was performed
